# Direct laser writing of 3D metallic mid- and far-infrared wave components

**DOI:** 10.1515/nanoph-2022-0604

**Published:** 2023-01-13

**Authors:** Erik Hagen Waller, Stefan Duran, Georg von Freymann

**Affiliations:** Fraunhofer Institute for Industrial Mathematics ITWM, 67663 Kaiserslautern, Germany; Physics Department and State Research Center OPTIMAS, University of Kaiserslautern, 67663 Kaiserslautern, Germany

**Keywords:** additive manufacturing, conductive structures, nanophotonics

## Abstract

A method for direct fabrication of 3D silver microstructures with high fabrication throughput on virtually any substrate is presented. The method is based on laser-induced photoreduction of silver ions to silver atoms, supported by nucleation, substrate functionalization and a multiple exposure fabrication process. The combination of the novel photosensitive suspension and the novel fabrication scheme enables effective fabrication speeds of up to 1 cm per second, with a minimum structure size of less than 1 μm, a resolution of more than 750 lines/mm and a resistivity of 3.0 · 10^−8^ Ωm. With this fabrication speed, it is now possible to produce conductive silver topographies several millimeters in length. Thus, with a single technology, one can fabricate photonic components with characteristic spectral features ranging from mid-to far-infrared.

## 1 Introduction

The mid- and far-infrared (IR) frequency range – often also referred to as the long-wave IR and terahertz region – extends from 300 GHz to 100 THz and is rich in applications such as non-destructive testing [1], imaging [2], and sensing in automotive applications [3]. Since this frequency range corresponds to wavelengths between 3 µm and 1 mm, the size of typical IR components, e.g., antennas also ranges from a few micrometers to several millimeters. Still, micrometer or even sub-micrometer precision is often required for the component to function as desired. Overall, this makes fabrication of functional metallic components working in the IR frequency range a challenging task for commonly employed microfabrication technologies. While photolithography as well as electron-beam-lithography are able to create two-dimensional structures, usually sample post-treatment processes like lift-off and/or chemical wet-etching are required to reveal the final metallic structure. Both techniques fail for fabricating three-dimensional structures. Additive manufacturing allows fabricating three-dimensional structures. However, most additive fabrication techniques address fabrication of millimeter-sized components working below 300 GHz [4] or only create templates, which have to be infilled with the desired metals and subsequently need removal. On the nanometer and few micrometer scale, some fabrication technologies that enable 3D metallic structures have been developed [5, 6]. While these techniques allow fabrication of highly resolved structures, they cannot cover larger areas or even reach the volume required for the frequency range addressed here. For this range, additive fabrication technologies are scarce. In this respect, direct laser writing (DLW) via multi-photon absorption is a very promising technology. In DLW, a photo initiator simultaneously absorbs two photons leading to polymerization in a well confined volume around the focal spot and thus DLW provides sub-micron feature sizes [7, 8]. Since polymerization takes place on the order of a few microseconds it allows for direct fabrication with speeds of several tens of centimeters per second [9, 10]. These high fabrication speeds, however, have so far only been obtained in the fabrication of polymeric structures. The fabrication of metallic structures (especially 3D topographies) has – up to now – been several orders of magnitude slower [11–13]. E.g., while we could show functional components working above 30 THz [14], fabrication speeds of 10 μm/s or less (0.5 μm^3^/s) prevented the application of this technology for cross-scale fabrication of mid- and far-IR components.

**Figure 1:**
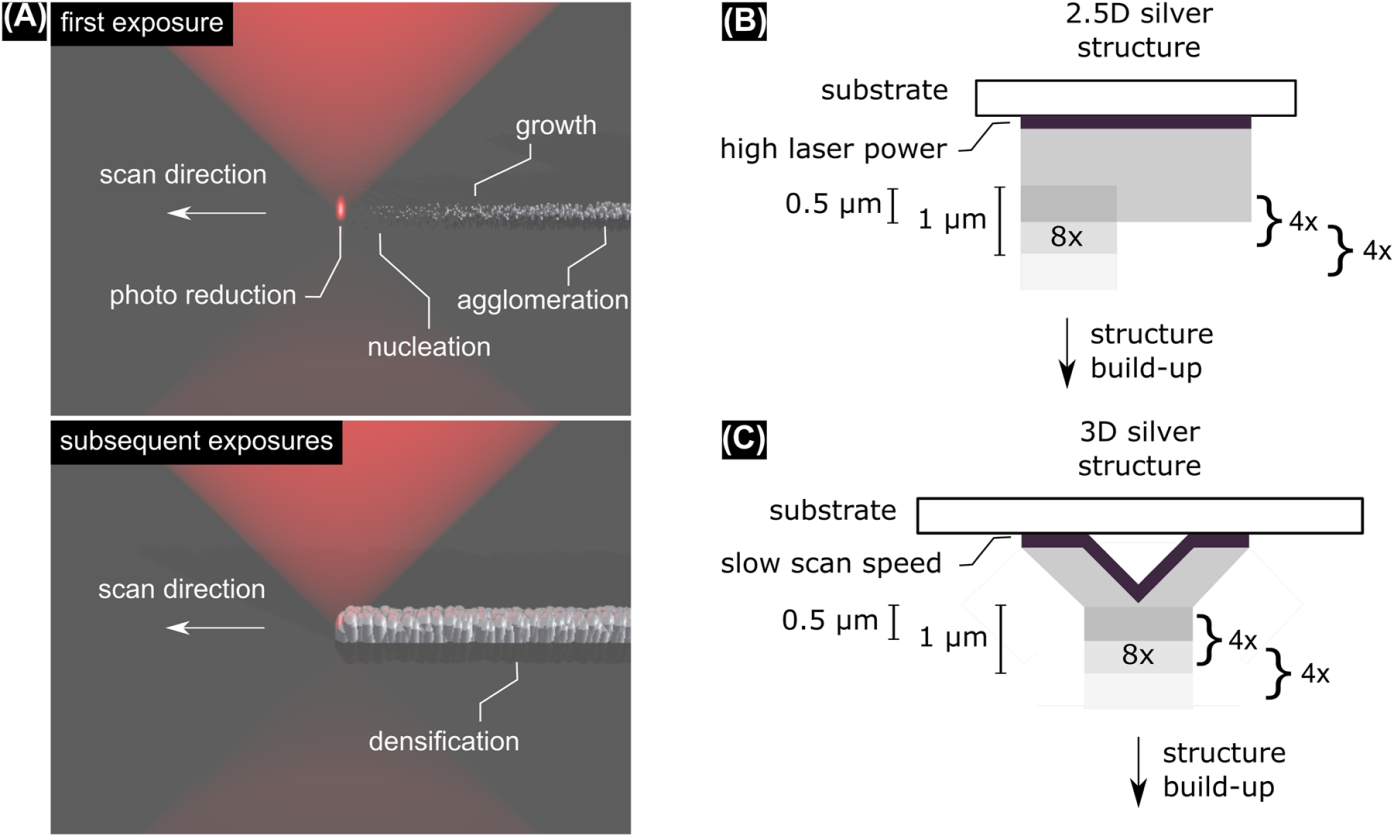
(A) Principle of DLW of metallic structures: after multi-photon excitation a photo reducing agent donates electrons to a silver precursor. The reduced silver ions nucleate to form seeds which grow to nanoparticles that subsequently agglomerate to form the building block of the structure. Subsequent exposures connect isolated agglomerates and densify the structure. (B) Fabrication strategy: a first seed layer is fabricated using high laser power. For subsequent layers laser power is reduced. Slices of 1 µm thickness are repeated four times before proceeding half a micrometer. Thus, each point is exposed 8×. (C) Fabrication scheme for 3D structures: The first overhanging layer is fabricated with slow scan speeds. All subsequent layers are fabricated following the same routine as in B.

The reason why DLW of metals is slow compared to DLW of polymers is due to the underlying structure formation process: in contrast to photopolymerization, DLW of metallic structures first involves photoreduction of metal ions, then nucleation of the metal atoms, growth of the nuclei, and finally agglomeration of the particles to the basic building block of the structure (see Figure 1A) [15]. This process occurs on the order of a few hundred milliseconds, orders of magnitude slower than polymerization [16, 17] and thus trails the laser beam. In principle, this process could be accelerated by increasing the incident laser power which would increase the number of seeds, lower the nucleation barrier and increase the nucleation and growth rates [18]. Why this is not readily possible is best explained by looking at the simplified threshold model [19]. In this model, the number of reduced metal atoms, *N*_red_, in the focal volume needs to exceed a certain threshold number, *N*_thr_, for nucleation and growth to occur sufficiently fast to enable connected structures. In two-photon initiated photo reduction this density is approximately proportional to the laser dose *D* [20]:

(1) $$N_{\text{red}}\propto D\propto \frac{{\left(P_{p}-P_{\text{thr}}\right)}^{2}}{v},$$

where *P*_p_ are incident pulse power, *P*_thr_ the threshold power and *v* the writing speed. To keep *N*_red_ constant above the threshold, an increase in *v* could be counteracted by increasing *P*_p_ as is usually done in photo polymerization. We recently, however, showed that structure-light interaction leads to increasing microbubbles due to boiling of the solvent when increasing the average intensity which is proportional to *P*_p_ [21]. One could simultaneously increase *P*_p_ and reduce the repetition rate by the same factor to avoid an increase in average intensity [22]. However, metal-light interaction is a one-photon process and thus the beneficial $P_{p}^{2}$ dependence of *N*_red_ turns to a linear dependence and therefore 3D topographies do not benefit from this.

Here, we present high-speed direct laser writing of silver microstructures on different substrates offering up to a centimeter per second fabrication speed, which – for 2.5D and 3D structures – is two orders of magnitude improvement compared to previous reports. The high fabrication speeds are obtained by using an improved photosensitive suspension, a novel fabrication strategy and optionally conjoined with surface functionalized substrates. We present the fabrication and evaluation of sample components with designed characteristic spectral features at 1.25 THz and 20 THz, proofing the applicability of the proposed method for cross-scale fabrication.

## 2 Materials and methods

As mentioned in the introduction, the fabrication strategy needs to take into account that we cannot simply ramp-up incident laser power to increase fabrication speed as is usually done in the fabrication of polymeric structures. The strategy employed here is presented in Figure 1B and is based on three pillars: substrate pretreatment, nucleation seed-assisted photo reduction and a multi-exposure fabrication process.

### 2.1 Photosensitive suspension

The abrupt change of optimal writing parameters such as laser power when structuring in previously unexposed parts versus in the vicinity of a structure is difficult to control [14]. Therefore, a high non-linearity in equation (1) is not necessarily beneficial. Still, photonic as opposed to thermal initiation of the photo reduction is desired. We found that introduction of silver nanoparticles with 60 nm diameter into a suspension attenuates this disruptive effect well. The nanoparticles show some residual one-photon absorption (see Supplementary material) and therefore lower the non-linearity in equation (1). This lowers the required laser power by a factor of two compared to a particle-free suspension. The particles furthermore function as nucleation seeds and compared to a previously published suspension increase the concentration of silver in the suspension [14]. Specifically, 10 wt% gelatin (gold grade, Carl Roth) is mixed with distilled water and left to bloom for 1 h. Subsequently, the suspension is heated for 3 h on a hotplate at 50 °C. In parallel, 0.4 M AgClO_4_ (monohydrate, Sigma-Aldrich) is added to 0.5 mL aqueous suspension of Ag nanoparticles (abcr GmbH, 60 nm diameter). The two suspensions are mixed in a 1:1 vol concentration and stirred for another hour at 50 °C on a hotplate using a magnetic stirrer. The colloidal suspension remains liquid when stored in a sealed bottle.

### 2.2 Substrate pre- and post-treatment

Since the presented photosensitive suspension is water-based, to achieve substrate independence, some substrates – e.g., hydrophobic substrates – benefit from pre-treatment. For example, we found that micro explosions sporadically occur close to a glass substrate surface without pre-treatment [14]. On the other hand, silicon wafers and silver or gold substrates do not need any pre-treatment due to their favorable surface energy. While there are numerous possibilities to achieve hydrophilic surfaces, we found a thin poly(dopamine) layer to work well on glass and polymer since it not only leads to hydrophilic surfaces and thus to good adhesion of silver on the substrate but also facilitates nucleation of photo reduced silver nanoparticles. Poly(dopamine) as an adhesion layer is well known and inspired by mussels. Hereby, spontaneous polymerization of dopamine leads to a thin layer of poly(dopamine) on almost any material [23–25]. In total, this layer leads to a higher probability of structure formation without the need to increase the average laser power and therefore avoids the above mentioned micro explosions. Substrates are first cleaned by subsequent immersion in an ultra-sonic acetone, isopropanol and distilled water bath for 10 min, respectively. Subsequently, substrates are blow-dried. Glass substrates are functionalized with poly(dopamine). To this end, the substrate is dipped into an aqueous solution of Tris-HCl (10 v%) and dopamine colorred (1 μg/mL) and left to rest for 48 h (air sealed). Subsequently, substrates are rinsed with water to remove excess poly(dopamine). The photosensitive suspension is sandwiched between the substrate and a glass window using tape as a spacer. To avoid evaporation of the water-based photosensitive suspension the construction space is sealed using glue. After exposure substrates are dipped into distilled water for 1 min to remove unexposed parts of the suspension and then left to dry.

### 2.3 Fabrication method

For direct laser writing a commercially available laser writer is used (Photonics Professional GT II, Nanoscribe). This system is based on a femtosecond pulsed laser with a wavelength of 780 nm and 80 MHz repetition rate. Unless otherwise stated, we use a 63× NA=1.4 immersion-oil objective (Zeiss) for focusing. Fabrication is done using the integrated galvanometer scanners with the scan speed given in the text.

Out of the three modifications, adaption of the fabrication process has the greatest impact on scan speed. Specifically, the fabrication process is adapted to account for the difference in required laser power when fabricating the first structure layer in unexposed regions compared to the subsequent layers by reducing the laser power for the latter (depicted in Figure 1B). The fabrication process is continued as follows: A slice with 1 µm thickness is hatched and sliced into lines and layers with 100 nm separation and exposed with a scan speed between 10,000 μm/s up to 80,000 μm/s. This cycle is repeated four times before adding a z-offset of 0.5 µm. By this sequence each point is exposed eight times and thus an effective scan speed of up to 10,000 μm/s (corresponding to 100 μm^3^/s) is reached, which is two to three orders of magnitude faster than scan speeds in previous reports. The advantage of the multi-exposure scheme is that in the first exposure seeds are created and their amount increased with higher efficiency by the following exposure steps. Therefore, despite the low efficiency of structure formation for single exposure, high scan speeds are possible without the need to increase laser power beyond damage threshold.

For overhanging 3D structures, a solid base layer is fabricated with a low scan speed of 1 μm/s up to 5 μm/s. On top of this first layer, the structure may then be built with the above mentioned high scan speeds (Figure 1C).

### 2.4 Four-point measurement

We use a home-built four-point measurement setup to measure the resistivity of fabricated structures. To this end, four equidistant pins are contacted with two electrodes which have been bridged by a fabricated beam with 10 µm × 10 µm × 50 µm (see Supplementary material). The voltage at the two inner pins is measured against the current at the two outer pins. The specific resistivity is then calculated using a thin wire model.

### 2.5 Fourier transform infrared spectroscopy

To measure mid-IR spectra of fabricated samples we use a Fourier transform infrared spectrometer (Bruker Vertex 70 V) and a microscope (Hyperion 3000) in reflection mode. A 15× Cassegrain objective and a KRS holographic wiregrid polarizer (Thorlabs) are employed to obtain the reflectance spectra. As reference a bare Si wafer is used.

### 2.6 Terahertz time-domain spectroscopy

In order to investigate the spectral sample characteristic in the terahertz frequency range between 0.1 THz and about 4 THz, a terahertz time-domain spectroscopy system (THz-TDS) is used in reflection geometry. In THz-TDS systems, optical pulses of a femtosecond laser source are split into an emitter and detector arm. To perform the pump-probe concept of a THz-TDS system, a fast moving mirror is placed in one arm to induce a phase shift between both laser pulses. Using GaAs-based photoconductive switches as emitter and detector antenna, the generated terahertz pulses can be measured with a high temporal resolution, and therefore, the amplitude and phase of the complex electric field are detected in time domain. With amplitude and phase, the spectral information of the terahertz pulse can be extracted using a Fourier transform [26]. The fabricated samples are investigated in a reflection geometry focusing the terahertz beam via two parabolic mirrors, respectively, onto the sample surface with a spot size of FWHM <2 mm. With the used system, we achieve a peak dynamic-range of more than 60 dB and a bandwidth of more than 4 THz at a measurement time of 1 s.

## 3 Results

**Figure 2:**
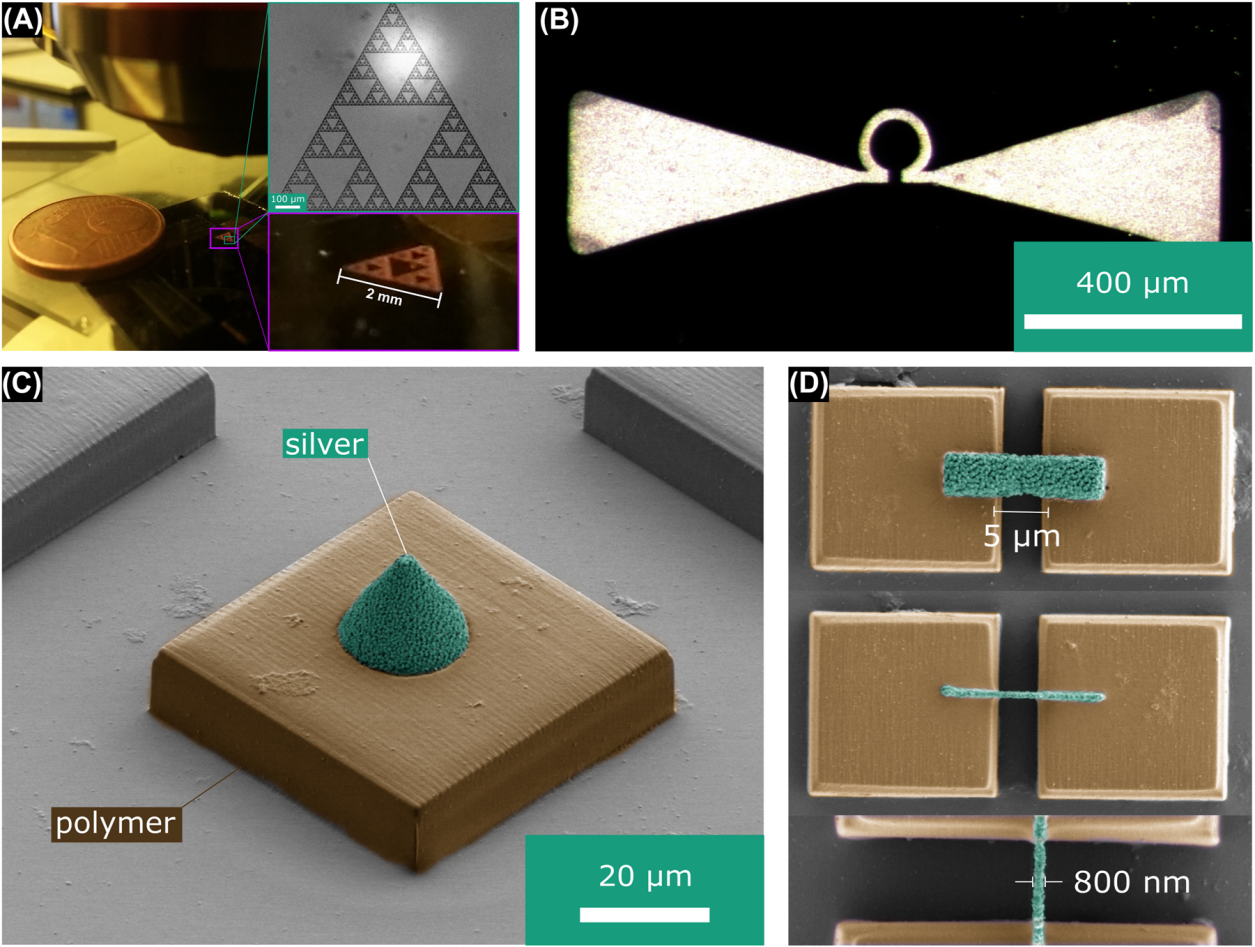
(A) Photograph of a Sierpinski-type fractal with 2 mm side length and a height of 2 µm on a silicon wafer. The insets show a reflection light microscopy image of one of the sub-triangles (top) and a zoom in on the photograph (bottom). (B) Magnified reflection light image of an omega-antenna type structure with 12 µm thickness. (C) Colorized SEM image showing a silver cone with 20 µm height (green) on top of a polymer block (brown). (D) Overhanging silver structures (green) bridging a 5 µm wide gap between polymer blocks (brown).

**Figure 3:**
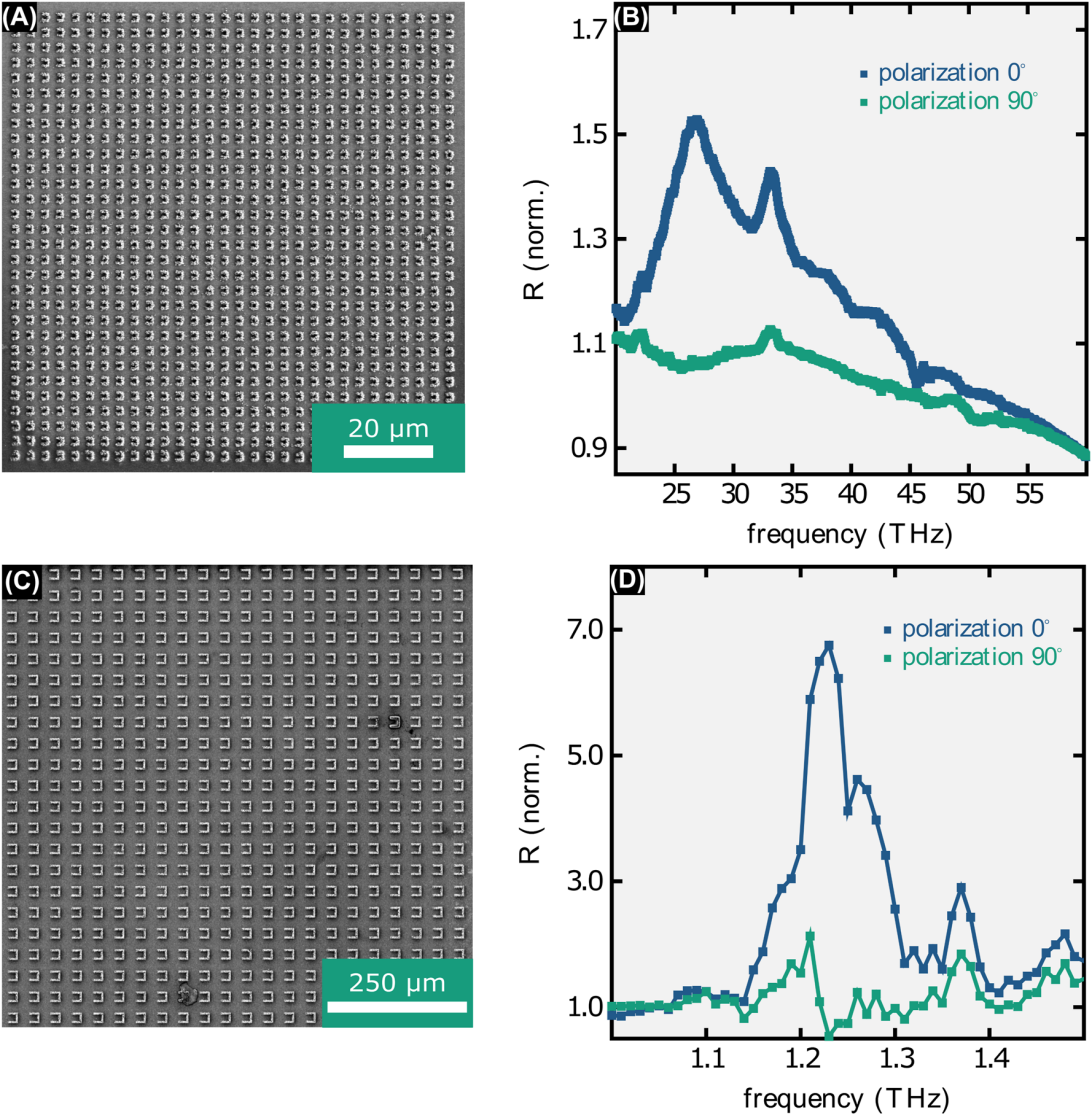
(A) SEM image of a split-ring-resonator array designed to reflect at 20 THz. (B) Corresponding mid-IR reflectance spectrum (normalized to reflectance of bare Si wafer). Due to fabrication tolerances the reflectance peak is shifted to approximately 27 THz. (C) SRR array designed to reflect at 1.25 THz. (D) Corresponding far-IR reflectance spectrum (normalized to reflectance of bare Si wafer, lines are a guide to the eye).

With the fabrication strategy described above it is now possible to fabricate silver topographies with a large height or on large areas as required by, e.g., many functional THz components. For example, in Figure 2A we show a photograph of a sample fractal structure that extends over several millimeters while maintaining a 2 µm line width. Fractals are, e.g., applied as antennas in mobile phones since they show several resonances and thus only one antenna may be used for GPS, wireless network and 5G frequency bands. While this structure is composed of thin lines in Figure 2B we show an omega-type structure fabricated with a 10× oil immersion objective which covers a large area. Similar structures are, e.g., used as antennas for the optical detection of magnetic resonances [13, 27]. This structure has a uniform height of 12 µm and sits on a non-conductive substrate. Structures with a thickness of several micrometers on non-conductive substrates are not easily fabricated by other techniques (such as photolithography and subsequent sputtering). While the above two structures are fabricated on silicon wafers, Figure 2C shows scanning electron micrograph images of a cone structure fabricated on an acrylic polymer block which indicates that almost any substrate may be chosen. Note, however, that aluminum reduces the silver ions and thus contact needs to be avoided. Finally, rapid fabrication of 3D structures is also possible (Figure 2D). Here, polymer blocks with a 5 µm wide gap are bridged by silver bridges. For these overhanging structures, the first layer or line was fabricated with 5 μm/s or 1 μm/s, respectively and subsequent layers or lines with 20,000 μm/s. To measure the resistivity of thus fabricated structures we fabricated silver beams measuring 10 µm × 10 µm × 50 µm connecting contact pads. The resistivity was then measured using a four-point measurement setup. It is interesting to note that the multi-exposure fabrication process leads to a very low resistivity of 3.0 · 10^−8^ Ωm, less than a factor of two higher than the resistivity of bulk silver (1.6 · 10^−8^ Ωm). This is two orders of magnitude lower compared to the resistivity of structures fabricated previously (approximately 10^−6^ Ωm) [14]. We attribute this firstly to the reduced influence of the structure surface since here the beams are large compared to the few micron thick wires used in the previous publication. The comparatively high beam volume reduces the impact of surface oxidation as well as the impact of small voids inside the structure. Secondly, we attribute the low resistivity to *in-situ* annealing that likely takes place during the multi-exposure fabrication process. The low resistivity enables the fabrication of functional component. Moving from sample structures to functional components we fabricated well-known split-ring-resonator (SRR) arrays on silicon wafers with one being designed to have resonant spectral features at 20 THz and the other at 1.25 THz (magnetic mode). The first marks the limit for the smallest details obtainable with the proposed method as the design leg-length is 1.32 µm, the design width 0.3 µm and the design height 0.75 µm. As visible in Figure 3A fabricated SRRs deviate by up to a few hundreds of nanometers from the design. Especially, the width is approximately 800 nm instead of the design width. Nevertheless, a broad resonance is visible in the reflectance spectrum for one polarization peaking at approximately 27 THz while it is absent in the other polarization, proving the principle functionality of the component (Figure 3B). The 1.32 µm separation of the SRR legs translates to a resolution of more than 750 dense lines/mm. The SRR array with its peak reflectance at 1.25 THz wavelength marks the upper limit of structures fabricated in a decent amount of time: while the individual SRR measures 16 µm in length, 3.5 µm in width and 3.5 µm in height (see Figure 3C) and thus is fabricated in less than a minute, to fill the area of the measurement spot (approximately 2 mm × 2 mm) of the THz-TDS system 3600 of those SRRs needed to be fabricated. Note, that without the here introduced method fabrication of such a structure would have taken more than half a year. Measurement results are shown in Figure 3C. As expected a clear reflectance peak close to design resonance frequency is visible for one polarization direction while it is absent at the other polarization direction.

## 4 Discussion and conclusion

We have presented a method to directly fabricate silver topographies with micrometer precision on almost arbitrary substrates. For 3D topographies, the fabrication speed achieved by this method is two to three orders of magnitude higher compared to previous works and now reaches scan speeds of up to 10 mm/s. While this method enables the fabrication of millimeter-sized structures with topography and micrometer precision that were up to now difficult to fabricate and despite the tremendous fabrication speed increase it is still one to two orders of magnitude slower compared to direct laser writing of polymeric structures. Furthermore, some difficulty arises from bending and detachment of structures during multi-exposure – it seems that heating and shrinkage need to carefully be balanced out. Despite these obstacles, we were able to demonstrate functional components, specifically split-ring-resonator arrays, with reflectance peaks in the mid- and far-infrared range, respectively.

## Supplementary Material

Supplementary Material Details
