# Supplementary material for: Direct laser writing of 3D metallic mid- and far-infrared wave components
Source: Nanophotonics. 2023 Jan 13;12(8):1549–55. doi: 10.1515/nanoph-2022-0604 (PMC11501879; doi:10.1515/nanoph-2022-0604)
Supplement: Supplementary file 1 — Supplementary Material Details [file j_nanoph-2022-0604_suppl.docx]

Erik H. Waller, Stefan Duran, Georg von Freymann

Supplementary

1.
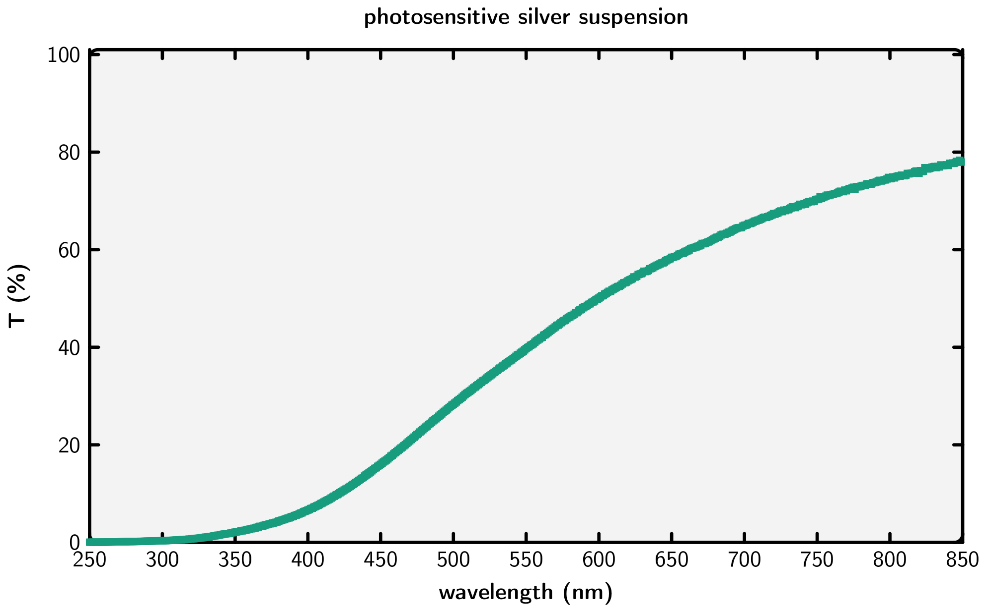
Transmittance spectrum of photosensitive suspension

Figure S 1: Transmittance spectrum of the final silver solution.

1. Conductivity measurements


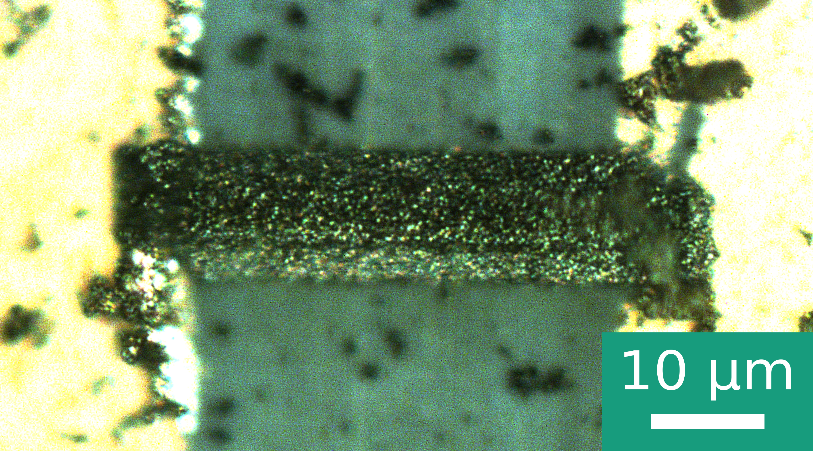
To conduct 4-point conductivity measurements first two electrodes were fabricated as follows: a Tollens reagent solution was exposed to UV light until a silver mirror formed. Using a razor blade, the thin silver layer was cut in two halves and electrical disconnection was verified. Between those electrodes a silver beam with 10 x 10 x 50 microns^3^ was fabricated using direct laser writing as shown in Figure S2. A home-built 4-point measurement device was used to measure the resistance of the thin silver layer alone (0.8 Ω) as well as the silver beam plus the silver layer (15.6 Ω). Using a thin wire model the resistivity was calculated by (R_beam+layer_ – R_layer_)*A_beam_/l_beam_, where A_beam_ is the area of the cross-section and I_beam_ is the length of the beam.

Figure S 2: Reflection light microscopy image of a direct laser written beam connecting two electrodes.
